# Latin-American Registry of Cardiovascular Disease and COVID-19: Rationale and Design of the CARDIO COVID 19-20 Registry

**DOI:** 10.5334/gh.925

**Published:** 2021-02-12

**Authors:** Juan Esteban Gómez-Mesa, Stephania Galindo-Coral, Maria Claudia Montes, Walter Alarco, Jose Luis Barisani, Antonio Magaña, Eduardo R. Perna, Alexander Romero, Mario Speranza, Iván Mendoza, Fernando Wyss

**Affiliations:** 1 Departamento de Cardiología de la Fundación Valle del Lili, CO; 2 Centro de Investigaciones Clínicas de la Fundación Valle del Lili, CO; 3 Instituto Nacional Cardiovascular INCOR – Essalud, PE; 4 Instituto Cardiovascular Adventista, Argentina. Hospital Presidente Perón, AR; 5 Asociación Nacional de Cardiólogos de México, MX; 6 Instituto de Cardiología JF Cabral, AR; 7 Hospital Santo Tomás, PA; 8 Hospital Clínica Bíblica, CR; 9 Instituto de Medicina Tropical de la Universidad Central de Venezuela, VE; 10 Servicios y Tecnología Cardiovascular de Guatemala S.A, GT; 11 Interamerican Society of Cardiology (IASC), MX; 12 Council of Heart Failure and Pulmonary Hypertension (CIFACAH) of the IASC, MX

**Keywords:** cardiovascular comorbidities, cardiovascular complications, COVID-19, Latin America

## Abstract

**Background:**

Infection caused by SARS-CoV-2 (severe acute respiratory syndrome coronavirus 2) exhibits a strong infectivity but less virulence compared to severe acute respiratory syndrome (SARS) and the Middle East respiratory syndrome (MERS). In terms of cardiovascular morbidity, susceptible population include elderly and patients with certain cardiovascular conditions. This infection has been associated with cardiac injury, cardiovascular complications and higher mortality.

**Objectives:**

The main objective of the CARDIO COVID 19-20 Registry is to determine the presence of cardiovascular comorbidities and cardiovascular complications in COVID-19 infected patients that required in-hospital treatment in different Latin American institutions.

**Methods:**

The CARDIO COVID 19-20 Registry is an observational, multicenter, ambispective, and hospital-based registry of patients with confirmed COVID-19 infection who required in-hospital treatment in Latin America. Enrollment of patients started on May 01, 2020 and was initially planned to last three months; based on the progression of pandemic in Latin America, enrollment was extended until December 2020, and could be extended once again based on the pandemic course in our continent at that moment.

**Conclusions:**

The CARDIO COVID 19-20 Registry will characterize the in-hospital population diagnosed with COVID-19 in Latin America in order to identify risk factors for worsening of cardiovascular comorbidities or for the appearance of cardiovascular complications during hospitalization and during the 30-day follow up period.

## Background

In December 2019, in Wuhan, China, various episodes of pneumonia caused by a new B-coronavirus (SARS-COV-2: Severe acute respiratory syndrome coronavirus 2) were identified and called Coronavirus Disease 2019 (COVID-19) by the World Health Organization (WHO) on February 11, 2020 [1]. This SARS-COV-2 has a zoonotic origin with a basic reproductive number or rate of contagion (R0) from 1.4 to 5.5 [2]. This high R0 led to a rapid expansion of COVID-19 cases in China, crossing borders to other continents, and leading to the declaration of the COVID-19 infection as a pandemic by the WHO on March 11, 2020 [3].

The main symptoms of this disease are fever (83-98%) and cough (76-82%). It is estimated that around 80% of cases are mild, 10%-15% are moderate, and up to 10% are severe and can present serious clinical manifestations, including cardiovascular and respiratory complications. Currently, several platforms record the number of cases by COVID-19 in a real and simultaneous way, such as Johns Hopkins University, USA. On March 27, 2020, this platform reported 558,502 confirmed cases, 25,251 deaths and 127,615 people recovered worldwide [1,4,5,6].

COVID-19 infection represented a health emergency in Europe, especially in Italy and Spain, and currently in the United States and Latin America. This health emergency has led different countries to implement rigorous political, economic, and social measures to prevent or delay the expansion of the SARS-COV-2 infection [7].

Pneumonia caused by SARS-CoV-2 exhibits strong infectivity, but less virulence compared to severe acute respiratory syndrome (SARS) and the Middle East respiratory syndrome (MERS). In terms of morbidity and mortality, susceptible population include elderly and patients with certain cardiovascular conditions. So far, treatments combined with powerful antiviral drugs, such as remdesivir, chloroquine, or lopinavir/ritonavir have shown confounding results. Based on that, more clinical trials are currently undergoing in order to determine the most specific transmission mechanisms, virulence, and pharmacological response [1].

Cardiovascular complications of influenza infection include myocarditis, acute myocardial infarction, and exacerbation of heart failure, well known during previous historical epidemics. Similarly, outbreaks of coronavirus have been associated with a significant burden of cardiovascular comorbidities and complications. Furthermore, the severity of the primary respiratory syndrome and the risk of adverse outcomes increases in patients with cardiovascular disease. Hypotension, tachycardia, bradycardia, arrhythmias, or even sudden death are common in patients with SARS [8].

In a retrospective multicenter cohort study of adult patients hospitalized with a confirmed diagnosis of COVID-19, of whom 137 were discharged and 54 died in hospital, high blood pressure was the most common comorbidity followed by diabetes mellitus and coronary heart disease. The multivariate regression showed higher probabilities of hospital death associated with older age, higher SOFA score (Sequential Organ Failure Evaluation Score) and D-dimer higher than 1 μg/ml on admission. The longest duration of virus clearance observed in survivors was 37 days [9].

The increased morbidity and mortality of COVID-19 in patients with high blood pressure have been observed in epidemiological studies in China. The association between Angiotensin-Converting Enzyme-2 (ACE-2) and SARS-CoV-2 infection have been described previously; ACE-2 acts as a co-receptor for the entrance of SARS-CoV-2 into the bloodstream. Based on this preliminary information, a concern related to the use of Angiotensin-Converting Enzyme inhibitors (ACEi) and Angiotensin-Receptor Blockers (ARB) in this population increased because the risk of higher severity and mortality in COVID-19. Observational studies showed no clinical evidence of cardiovascular adverse outcomes with the use of these drugs and a recent randomized clinical trial confirmed this finding [10,11]. The Council of Hypertension of the European Society of Cardiology and other international societies published different statements clarifying this topic and recommended continuing cardiovascular treatment with these drugs [12,13].

## Introduction

In February 25 of 2020, the first case of COVID-19 was confirmed in Latin America. This first case occurred in South America and was a 61 year-old Brazilian man who had traveled to Lombardy, Italy (from February 9 to February 20) [14]. The first case confirmed in Central America occurred on February 28 in Mexico, and was a 35 year-old man who had also traveled to Italy in February [15]. Following that, all countries on the American continent have reported positive cases. Considering the high prevalence of cardiovascular disease in the countries of Latin America and its direct relationship with mortality, it is expected that the current COVID-19 pandemic may negatively impact this population more severely.

Based on these factors, it is necessary to carry out a multicenter registry of in-hospital patients with COVID-19 that will allow determination of the relation of baseline characteristics, cardiovascular comorbidities, and cardiovascular treatments with the risk of cardiovascular complications and worse outcomes in patients during hospitalization and during the 30-day follow up.

## Methods and Design

### Study population

The study population will include hospitalized patients with confirmed COVID-19 infection, with or without previous cardiovascular comorbidities.

### Inclusion criteria

- Patients older than 18 years with confirmed diagnosis of COVID-19 according to guidelines given by the World Health Organization and according to each institutional and/or local guideline, and
- who requires in-hospital management for more than 24 hours related to COVID-19, or
- who dies during the first 24 hours after hospital admission

### Informed Consent

Informed consent is not required because this is an observational study. If any institution requires informed consent, a form that was validated by the CIC and the Ethics Committee of the FVL is available and can be used.

### Study design

The CARDIO COVID 19-20 Registry is an observational, multicenter, ambispective, and hospital-based registry that includes patients with confirmed COVID-19 that require in-hospital treatment in Latin American medical institutions. Enrollment of patients started on May 01, 2020 and was initially planned to last three months; however, based on the progression of the pandemic in Latin America, enrollment was extended until December 2020, and could be extended once again based on the pandemic course in this continent at that time.

The Scientific Committee is composed by members of the Council of Heart Failure and Pulmonary Hypertension (CIFACAH) and the Interamerican Society of Cardiology (IASC). It has the approval of the Clinical Research Center (CIC) and the Ethics Committee of the Fundación Valle del Lili (FVL) and the Scientific and Academic approval of IASC.

### Site selection

**Figure 1:**
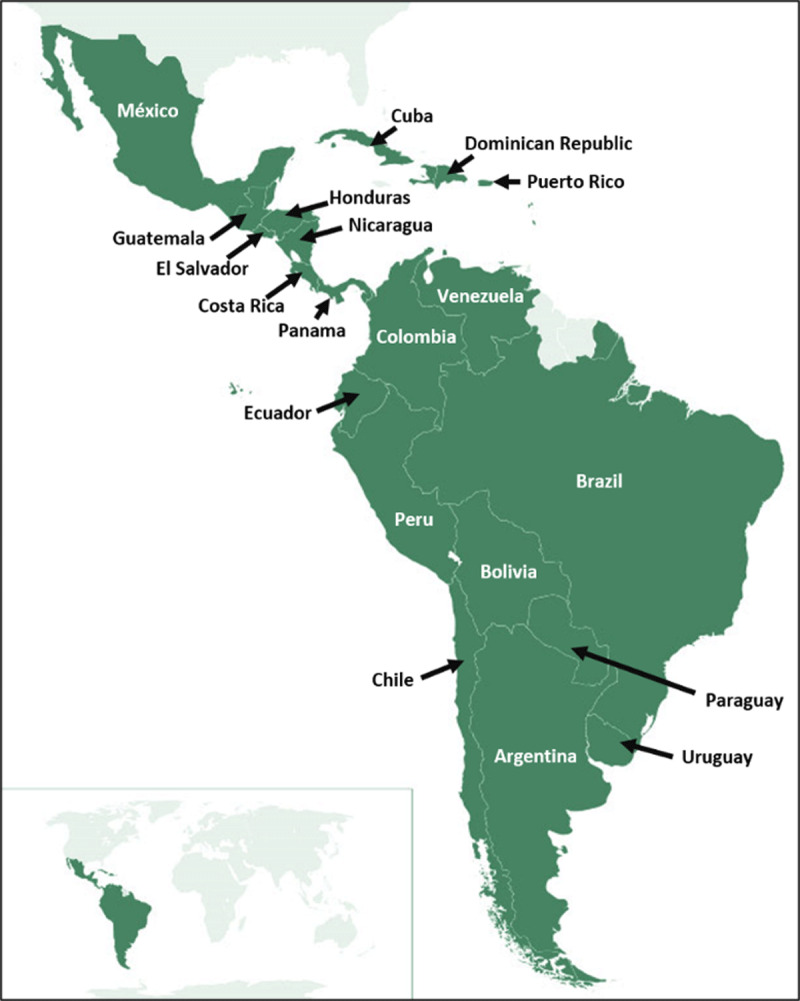
Participant countries in Central and South America.

The Steering Committee of the Registry designated a national coordinator in the 20 Latin American countries that will be included; from Central America: Mexico, Guatemala, Honduras, El Salvador, Nicaragua, Costa Rica, Panama, Dominican Republic, Cuba and Puerto Rico; and from South America: Colombia, Venezuela, Brazil, Ecuador, Peru, Bolivia, Uruguay, Paraguay, Chile and Argentina. (Figure 1) The IASC and CIFACAH are working with those designated national coordinators and the presidents of the national cardiovascular societies or associations of these countries (who must be affiliated to the IASC) to identify potential researchers (e.g., internal medicine specialists, cardiologists or intensivists) that will be contacted and invited to participate in this project. Those identified professionals and institutions must complete a digital form submitting data to the research team (principal investigator and sub investigator).

## Data Collection

Information (variables) will be collected in the electronic database system RED Cap (Research Electronic Data Capture: https://www.project-redcap.org/). This database system will include 277 variables that will be distributed in 15 different files (Table 1).

**Table 1:** Information to be collected.

Information	Variables

Demographics	*Age, gender, and other variables that will allow this population to be characterized*
Comorbidities	*Mainly cardiovascular pathologies including hypertension, coronary artery disease, heart failure and dyslipidemia, among others. Other high-risk comorbidities like cancer, immunosuppressive conditions, obesity, diabetes mellitus, COPD and renal failure will be included*.
Previous cardiovascular treatment	*ARB/ACEi/ARNI, and other drugs like beta blockers, diuretics, antiplatelet and anticoagulant agents*
Symptoms at admission	*Includes the most frequently reported (fever, cough, chest pain, and fatigue) and other less frequent variables (dysphagia, loss of taste or smell, myalgias, diarrhea…)*
Clinical findings at admission	*Basic hemodynamic and metric evaluation*
Cardiovascular complications during hospitalization	*Myocarditis, arrhythmias, heart failure, coronary artery events, venous or arterial thrombosis*
Laboratory test at admission	*Complete blood count, kidney and liver function tests, cardiovascular biomarkers and inflammatory biomarkers*
Electrocardiography	*Rhythm, QRS complex, bundle, branch block, corrected QT interval*
Chest X-Ray	*Pulmonary infiltrates, cardiomegaly, lung congestion, pleural effusion*
Echocardiography	*RV systolic function, pericardial effusion, cardiac tamponade, valvular function, vena cava dilatation, measurement of LV size and LV global systolic function*
Cardiovascular procedures performed during hospitalization	*Central catheters, use of inotropes, vasopressors or vasodilators, mechanical ventilation, ventricular assist devices (IABP, ECMO)*
COVID-19 treatment during hospitalization	*Steroids, antivirals, anti-inflammatory, anticoagulant or any other used treatment*
Laboratory tests at discharge	*Complete blood count, kidney and liver function tests, cardiovascular biomarkers and inflammatory biomarkers*
Outcomes during hospitalization and at discharge	*Length of stay, condition at discharge (alive or dead)*
Outcomes 30 days after discharge	*Rehospitalization, condition at follow up (alive or dead)*

COPD: Chronic Obstructive Pulmonary Disease; ARB: Angiotensin-Receptor Blockers; ACEi: Angiotensin-Converting Enzyme Inhibitors; ARNI: Angiotensin Receptor Neprilysin Inhibitor; RV: Right Ventricle; LV: Left Ventricle; IABP: Intra-Aortic Balloon Pump; ECMO: Extracorporeal Membrane Oxygenation.

Each institution will be coded, as well as the identity of every enrolled patient. Weekly quality control will be carried out in each participant center in order to verify the integrity of the data collected and will be informed about discrepancies found in the registered data.

## Data Analysis

This study will obtain different data that will be used to calculate lethality and frequency of complications during hospitalization and follow-up, and to characterize the overall in-hospital population. Hospital fatality (number of deaths attributed by COVID-19/number of patients diagnosed with COVID-19), incidence of in-hospital cardiovascular complications (number of new complications due to specific cardiovascular cause in COVID-19 patients/number of patients diagnosed with COVID-19), and survival analysis (the probability of death by time interval can be calculated) will be determined by the Kaplan Meier method and presented by a graph.

Characterization of the in-hospital population will be carried out through an univariate analysis to determine the behavior of numerical variables, the normality of the variables will be determined with a Shapiro Wilk test, those with a p > 0.05 will be considered with normal distribution and will be presented with averages and standard deviation; those with a non-normal distribution will be presented with median and interquartile ranges. The categorical variables will be presented as proportions.

The quantitative variables will be compared, in case of a normal distribution, by the Student’s t-test or ANOVA. If the normality criterion is not met, a non-parametric test will be used. For the comparison of categorical variables, Fisher’s exact test or X^2^ test will be used. For the analysis of patient outcomes with COVID-19 based on demographic, laboratory values, and/or clinical variables, the relation of these variables will be evaluated with a multivariate logistic or linear regression models, depending on the outcome variable to evaluate.

## Conclusion

The real impact of the COVID-19 pandemic in the Latin American population is unknown. This registry will allow characterization of the population with this infection to determine the risk of complications in patients with cardiovascular comorbidities; to describe de-novo cardiovascular complications, in-hospital mortality, and 30-day follow up outcomes.
